# Bispidine-Amino Acid Conjugates Act as a Novel Scaffold for the Design of Antivirals That Block Japanese Encephalitis Virus Replication

**DOI:** 10.1371/journal.pntd.0002005

**Published:** 2013-01-17

**Authors:** V. Haridas, Kullampalayam Shanmugam Rajgokul, Sandhya Sadanandan, Tanvi Agrawal, Vats Sharvani, M. V. S. Gopalakrishna, M. B. Bijesh, Kanhaiya Lal Kumawat, Anirban Basu, Guruprasad R. Medigeshi

**Affiliations:** 1 Department of Chemistry, Indian Institute of Technology, New Delhi, India; 2 Vaccine and Infectious Disease Research Center, Translational Health Science and Technology Institute, Gurgaon, India; 3 National Brain Research Center, Manesar, Haryana, India; Institute of Tropical Medicine (NEKKEN), Japan

## Abstract

**Background:**

Japanese encephalitis virus (JEV) is a major cause of viral encephalitis in South and South-East Asia. Lack of antivirals and non-availability of affordable vaccines in these endemic areas are a major setback in combating JEV and other closely related viruses such as West Nile virus and dengue virus. Protein secondary structure mimetics are excellent candidates for inhibiting the protein-protein interactions and therefore serve as an attractive tool in drug development. We synthesized derivatives containing the backbone of naturally occurring lupin alkaloid, sparteine, which act as protein secondary structure mimetics and show that these compounds exhibit antiviral properties.

**Methodology/Principal Findings:**

In this study we have identified 3,7-diazabicyclo[3.3.1]nonane, commonly called bispidine, as a privileged scaffold to synthesize effective antiviral agents. We have synthesized derivatives of bispidine conjugated with amino acids and found that hydrophobic amino acid residues showed antiviral properties against JEV. We identified a tryptophan derivative, Bisp-W, which at 5 µM concentration inhibited JEV infection in neuroblastoma cells by more than 100-fold. Viral inhibition was at a stage post-entry and prior to viral protein translation possibly at viral RNA replication. We show that similar concentration of Bisp-W was capable of inhibiting viral infection of two other encephalitic viruses namely, West Nile virus and Chandipura virus.

**Conclusions/Significance:**

We have demonstrated that the amino-acid conjugates of 3,7-diazabicyclo[3.3.1]nonane can serve as a molecular scaffold for development of potent antivirals against encephalitic viruses. Our findings will provide a novel platform to develop effective inhibitors of JEV and perhaps other RNA viruses causing encephalitis.

## Author Summary

Japanese encephalitis virus is a mosquito-borne virus, which causes encephalitis primarily in children and is a major cause of encephalitis-related deaths in South and South-East Asian countries. Although new and safe vaccines are available for use, it is neither affordable nor readily available in endemic regions. Currently there are no antivirals for JEV treatment and developing new drugs against JEV is the need of the hour. In this study we used the backbone of the naturally occurring lupin alkaloid, sparteine to synthesize derivatives containing amino acid residues. We found that the conjugate consisting of the hydrophobic amino acid tryptophan, Bisp-W, inhibited virus replication in cell culture studies. We show that Bisp-W inhibits virus infection at a stage post-entry and at the level of viral RNA replication suggesting that this compound could serve as a potential therapeutic option for treating JEV infection. We believe that the chemical scaffold identified in this study could serve as a platform for synthesizing more potent antivirals that could be used to treat viral encephalitis.

## Introduction

Japanese encephalitis virus (JEV) is a mosquito-borne, neurotropic RNA virus within the family *Flaviviridae*, which includes other pathogens of global significance such as dengue virus and West Nile virus. JEV is endemic to most South- and South-East Asian countries and recurring outbreaks lead to about 30,000 deaths, mostly children, annually. Patients recovering from JEV infections suffer from long-term neurological sequelae. Availability of WHO-approved JEV vaccine for pediatric use is limited and there are no clinically approved antivirals for JEV infections. JEV genome is a single-stranded positive strand RNA encoding for a single poly-protein that is cleaved by host and viral proteases into three structural (Capsid, precursor membrane/membrane and envelope) and seven non-structural proteins (NS1, 2A, 2B, 3, 4A, 4B and 5). Pigs and water birds have been proposed to be the natural hosts of JEV and Culex mosquitoes transmit the disease to humans who are dead-end hosts [1], [2].

Protein-protein interactions are vital to many cellular events and blocking such interactions using synthetic compounds is a very attractive option for design of new pharmaceuticals. The formation of homomeric and heteromeric protein complexes involves very stringent selection based on chemical complementarity. Therefore targeting such interaction requires a structure-based approach. The therapeutic utility of secondary structure peptide mimetics is under intense investigation particularly to treat cancer and HIV and also to serve as antimicrobial compounds [3]–[5]. Protein secondary structure based inhibitors are one of the rational ways in achieving this goal. In this study, we describe 3,7-diazabicyclo[3.3.1]nonane, commonly called bispidine, as a privileged scaffold for generating potential antiviral agents. We chose bispidine moiety, because of its rigidity and high hydrophobic surface area, which enables interaction with hydrophobic surfaces of cellular/viral proteins and thus may alter the profile of protein interaction network. We envisioned that, when appended with amino acids, bispidine scaffold can orient the amino acids in rigid geometrical arrangement and thus would be an ideal scaffold to generate various ligands for inhibiting protein-protein interactions [6]. We validated this hypothesis by using JEV as a model and show that one of the derivatives of bispidine exhibits potent inhibitory activity against JEV replication suggesting that bispidine can be used to develop novel therapeutics against flaviviruses.

## Materials and Methods

### Cell lines and virus

Mouse neuronal cells, Neuro2A (N2A) and human hepatoma cells (Huh7) were grown at 37°C in Dulbecco's minimum essential medium (DMEM) containing 10% fetal bovine serum, penicillin, streptomycin and non-essential amino acids. C6/36 cells were grown at 28°C and Vero cells were grown at 37°C in minimum essential medium with Earl's salts and additives as above. JEV-Vellore strain passaged in mouse brain was kindly provided by Sudhanshu Vrati. West Nile virus (strain 68856) was obtained from the National Institute of Virology, Pune. Chandipura virus (strain 1653514) was kindly provided by Dhrubajyoti Chattopadhyay. JEV was passaged once in porcine kidney cells (PS) and virus stocks thus obtained were stored at −80°C. PS cell-derived virus was used throughout this study. West Nile virus stocks were prepared in C6/36 cells. Chandipura virus was passaged once in Vero cells for generation of viral stocks. Viral titers were measured using plaque assays as per our previously established protocols for West Nile virus except that the plaque assays for JEV were performed on PS cells instead of Vero cells [7]. For Chandipura virus, plaque assays were performed on vero cells and samples were incubated for 12 hours and fixed as described above.

### Virus infection, compound treatment

Cells were grown in 24-well or 48-well plates to about 70% confluence. Cells were infected with JEV at the multiplicity of infection (MOI) of 1 plaque forming unit (pfu)/cell. For inhibition experiments, cells were treated at indicated time-points with indicated concentrations of bispidine derivatives, washed twice with phosphate buffered saline (PBS) and incubated with virus medium (DMEM containing 2% fetal bovine serum with additives) containing the required amount of virus or no virus (uninfected controls) for one hour on a rocking platform in CO_2_ incubator at 37°C. After incubation, virus inoculum was removed and cells were washed twice with PBS and fresh medium (DMEM-2% fetal bovine serum with additives) containing the indicated concentration of compound/s or equal volume of DMSO was added onto the cells and continued incubation for indicated periods. Minocycline treatment was performed as described previously [8], briefly, cells were pre-treated for 1 h with either minocycline (20 µM) or Bisp-W (5 µM) and corresponding vehicle controls (PBS/DMSO). Cells were infected as above in the absence of compounds and compounds were added after virus adsorption. Culture supernatants were collected and viral titers were measured using plaque assays. Intracellular virus titers were measured by collecting cells by trypsinisation and resuspending cells in PBS. Cell suspension was subjected to three freeze-thaw cycles by placing in liquid nitrogen and 37°C water-bath consecutively in each cycle. Supernatants were collected by centrifugation at 3,000 rpm for 5 min. at 4°C and used in plaque assays as described above. For West Nile virus inhibition studies, cells were infected with an MOI of 1 pfu/cell and supernatants collected at 22 hours post-infection (h pi) and for Chandipura virus, cells were infected with an MOI of 0.1 pfu/cell and supernatants collected at 8 h pi. Viral titers were estimated by plaque assay on Vero cells as described above.

### Cytotoxicity assay

Cells were infected as described above and cytotoxicity was assessed by lactate dehydrogenase release assay using CytoTox 96 Non-Radioactive Cytotoxicity Assay kit as per the manufacturer's instructions (Promega catalog no. G1780).

### Quantitative real time PCR

Total cellular RNA was prepared from JEV-infected and Bisp-W-treated cells using Trizol as per manufacturer's instructions (Invitrogen). 100 ng of total RNA was used to estimate JEV genome copy numbers using taqman one-step real time PCR reagents (Applied Biosystems). PCR conditions were as follows: 48°C–30 min., 95°C–10 min. followed by 40 cycles of 95°C–15 sec., 60°C–1 min. Standard curve was generated using in vitro transcribed JEV-NS3 from a plasmid clone. Viral genome copy numbers were estimated using NS3 primer and probe set described previously [9]. GAPDH was used for normalization of the samples (Forward primer: 5′- TGTGTCCGTCGTGGATCTGA - 3′ Reverse primer: 5′- CCTGCTTCACCACCTTCTTGA - 3′ Taqman probe: 5′-FAM-CCGCCTGGAGAAACCTGCCAAGTATG-TAMRA).

### Western blot analysis

N2A cells were infected with JEV and treated with Bisp-W as above. 22 h pi, cells were washed twice in PBS on ice and lysates were prepared in lysis buffer containing Tris-HCl (50 mM, pH 8.0), 150 mM sodium chloride, 1% IGEPAL CA-630, 0.5% sodium deoxycholate, 0.1% SDS and protease inhibitor cocktail (Roche). Equal amount of protein was resolved on a 15% SDS-PAGE and transferred onto Immobilon PVDF membrane (Millipore) and probed with rabbit polyclonal antibodies raised against JEV-capsid protein using goat-anti-rabbit horseradish peroxidase-conjugated secondary antibody (Millipore). Blots were developed using chemiluminescence substrate (Thermo Scientific Pierce).

### Immunofluorescence

N2A cells were grown on cover slips and infected with JEV and treated with BLB as described above. 24 h pi cells were washed with cold PBS twice and fixed with cold methanol at −20°C for 20 min. Cells were washed twice with PBS and PBS containing 0.2% BSA (PBS-BSA) followed by incubation with primary (4G2-Millipore) and secondary (donkey anti-mouse IgG conjugated with Alexa 546 dye) antibodies for one hour each at room temperature. Cells were washed after respective antibody incubations with PBS-BSA three times each and coverslips were mounted on glass slides with Prolong anti-fade reagent (Invitrogen). Images were acquired by using 40× oil immersion objective in Olympus FLUOVIEW FV1000 confocal microscope.

### Statistical analysis

GraphPad Prism software was used for all graphical representations and statistical analysis. Significance of inhibition was estimated by non-parametric, unpaired, two-tailed t-test.

### Compound synthesis and purification

Detailed procedure for compound synthesis, NMR and HPLC profile of some of the compounds used in the study is provided as supplementary information, Methods, S1.

## Results

### Design rationale and synthesis of bispidine conjugates

**Figure 1:**
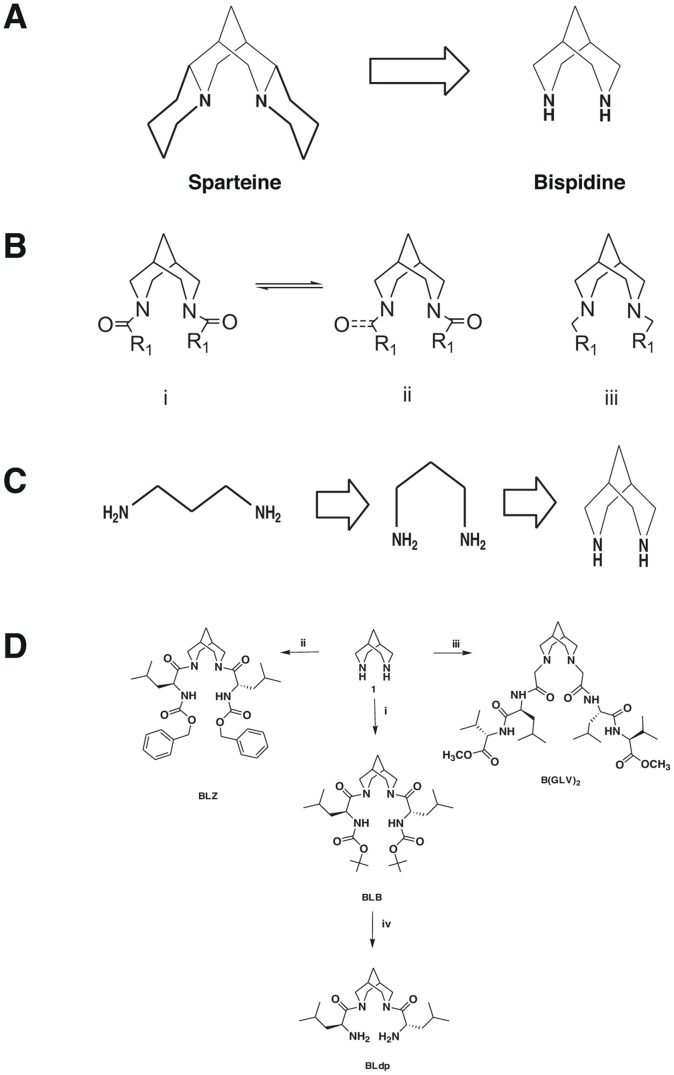
Bispidine and its derivatives. (A) Structural relationship of bispidine to natural product sparteine. (B) General structure of bispidine compounds (i) and (ii) represent the conformer equilibrium in diimides of bispidine (iii) Conjugates of bispidine wherein the nitrogen is part of the amino acids. (C) The genesis of design of bispidine. (D) Synthesis of bispidine conjugates i) N-hydroxysuccinimide/DCC/NEt_3_/Boc-Leu-OH/dry dichloromethane; ii) N-hydroxysuccinimide/DCC/NEt_3_/Z-Leu-OH/dry dichloromethane; iii) Br-CH_2_-CO-Leu-Val-OMe; NEt_3_, acetonitrile; iv) 25% TFA in dichloromethane.

Bispidine unit is the backbone of the naturally occurring lupin alkaloid, sparteine (Figure 1A). The rigid bicyclic molecular framework of bispidine can be considered as a constrained amino acid surrogate (diaminopropane derivative). The presence of two nitrogen at a distance ∼3 Å is an ideal situation to functionalize with amino acids or peptides. The bicyclic framework coupled with closely spaced nitrogen atom ensures a turn conformation. Interestingly the imide carbonyls can have free rotation and thus may exist in syn- or anti- form thereby mimicking proline. The U architecture and the closely spaced nitrogen atom ensure a turn-like structure on the attached peptides (Figure 1B and 1C). The rotational freedom of the occurrence of two form (i) and (ii) mimic proline-like environment and expected to nucleate or favor a particular secondary structure. The NMR and X-ray crystallographic evidence propose a sheet-like structure for diimide derivative. We envisaged that the bispidine moiety would be an ideal scaffold to maintain the peptide chains in parallel or antiparallel fashion to induce a specific conformation. The closely positioned nitrogen atoms could be used to induce conformational changes via protonation.

The bispidine framework was synthesized by a double Mannich reaction involving Boc-piperidone, benzylamine, formaldehyde and acetic acid [10]. The resulting bispidinone was reduced to yield a bispidine derivative. The benzyl and boc protecting groups in bispidine allowed selective functionalization of the two nitrogen atoms. The leucine-anchored derivative BLB was synthesized by coupling *t*-butyloxylcarbonyl protected leucine (Boc-Leu-OH) with bispidine using N-hydroxysuccinimide and dicyclohexylcarbodimide (DCC). Similarly, benzyloxycarbonyl protected leucine (Z-Leu-OH) yielded BLZ. Deprotection of BLB using 25% TFA in dichloromethane yielded BLdp. We also synthesized hexapeptide derivative of bispidine B(GLV)2, wherein the amino acid nitrogen is part of the bispidine moiety (Figure 1D). Similarly all the analogues were synthesized by reacting protected amino acid derivatives with bispidine using DCC coupling procedure.

### Effect of bispidine conjugates on JEV production

**Figure 2:**
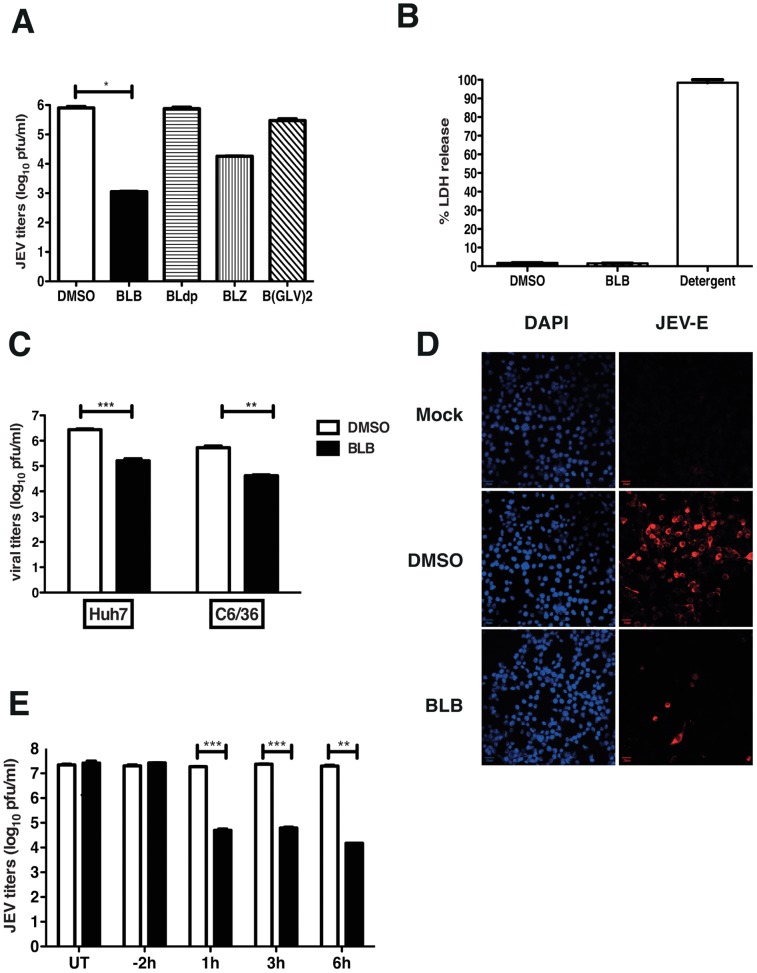
Effect of Bispidine derivatives on JEV infection. (A) Viral titers were determined by plaque assay of N2A cell culture supernatants (22 h pi) infected with JEV and treated with 100 µM of derivatives of bispidine. * P<0.01 determined by two-tailed, t-test. (B) Cytotoxicity was measured by lactate dehydrogenase (LDH) assay from culture supernatants treated with 100 µM of BLB or DMSO. LDH released from cells incubated with detergent buffer was used as 100% LDH release. (C) Viral titers were determined by plaque assay from Huh7 and C6/36 cell culture supernatants (22 h pi) infected with JEV and treated with 100 µM of BLB. *** P = 0.0002 and **P = 0.0041 determined by two-tailed, t-test. (D) N2A cells were infected as above and at 22 h pi cells were fixed and stained with anti-E antibodies followed by alexa 568-conjugated secondary antibodies. Nuclei were stained by DAPI. (E) Viral titers were determined by plaque assay of N2A cell culture supernatants (22 h pi) infected with JEV and treated with 100 µM of BLB at the indicated time points. UT- Untreated. All the data presented are representative of two or more experiments performed with two or more replicates. *** P = 0.0007, 0.0005 and **P = 0.007 as determined by two-tailed, t-test. Error bars in all figures represent Mean ± SEM.

We tested for the anti-viral property of bispidine-conjugates using JEV replication model in N2A cells. Cells were infected with JEV and after the adsorption of the virus, cells were grown in medium containing bispidine conjugates and at 22 h pi viral titers in the infected cell supernatants were measured by performing plaque assays. Cells treated with the leucine derivative, BLB, consistently reduced viral titers by hundred to thousand-fold compared to cells treated with DMSO. The removal of N-protecting groups (BLdp) led to complete loss of anti-JEV activity. Changing the terminal group from a *t*-butyloxycarbonyl group (BLB) to benzyloxycarbonyl (BLZ) on the terminal of leucine lowered the viral inhibition efficiency while the hexapeptide derivative (B(GLV)2) showed a decrease in anti-JEV activity indicating the dependence on the nature of the amino acid unit on the inhibitory properties of these compounds (Figure 2A). We next assessed if the observed effect of BLB on JEV titers is due to reduced cell numbers as a result of cell death. BLB treatment was found not to affect cell viability (by trypan blue exclusion, data not shown) or induce cytotoxicity at the concentration used for viral inhibition (Figure 2B). JEV infects a wide-variety of species and cell type, therefore, we next tested whether BLB was capable of inhibiting JEV replication in cell lines from different species. Huh7 and C6/36 cells were infected with JEV and treated with BLB as mentioned above. We found that BLB inhibited JEV infection by more than ten-fold in both these cell lines indicating that the target of BLB could be one of the viral protein/s or a host factor which is conserved across species and which plays a role in JEV replication (Figure 2C). The inhibitory effect in these cell lines appears to be less than that observed in N2A cells suggesting that the cells of neuronal origin are probably more sensitive to the action of BLB. Previous reports of JEV inhibition using peptide-conjugated morpholino oligomer also showed a similar cell-type dependent effect [11].

### BLB acts at a stage prior to viral protein translation and post viral entry

In the experiments described above, BLB was added on to the cells after virus adsorption, as our objective was to identify inhibitors that block JEV infection at post-entry stages. Immunofluorescence analysis of JEV-infected, BLB-treated cells revealed very few infected cells compared to DMSO treatment (Figure 2D) suggesting that the effect of BLB on JEV production is at a stage prior to viral protein translation. We next performed time-of-addition experiments wherein cells were treated with BLB for 2 h prior to virus infection or at indicated times post-infection and viral titers in the supernatant were measured by plaque assays. Pre-treating cells with BLB had no effect on viral titers whereas addition of BLB at 1, 3 or 6 h post-infection effectively blocked viral titers to the same extent suggesting that the effect of BLB is at a stage post-viral entry and capsid disassembly (Figure 2E) indicating that BLB possibly affects JEV viral genome replication.

### Synthesis of bispidine conjugates with enhanced antiviral activity

**Figure 3:**
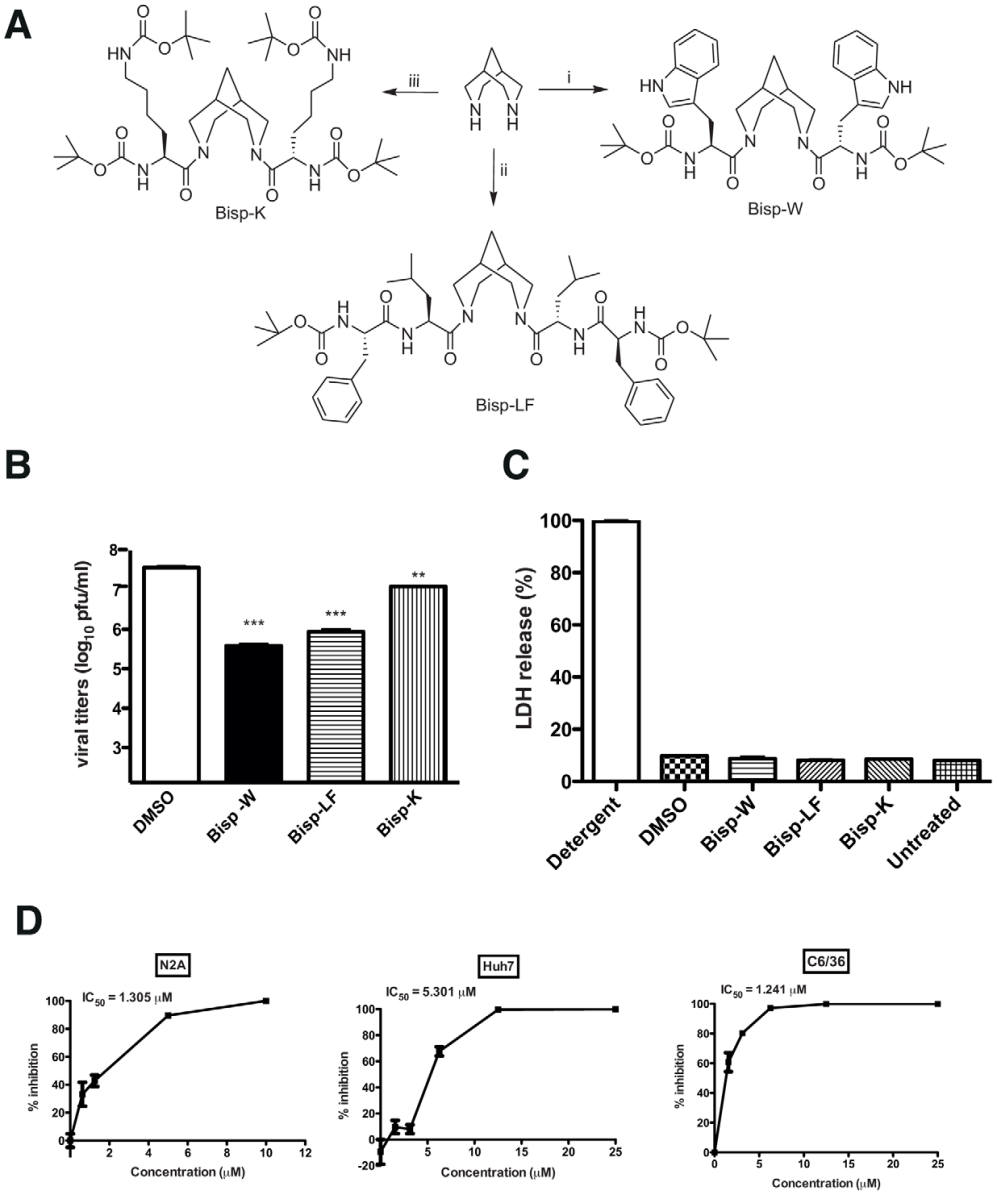
Synthesis of amino acid conjugates of bispidine. (A) Structure of bispidine conjugated with tryptophan (Bisp-W), lecine+phenylalanine (Bisp-LF) and lysine (Bisp-K). (B) Viral titers were determined by plaque assay of N2A cell culture supernatants (22 h pi) infected with JEV and treated with 5 µM of derivatives of bispidine. *** P = 0.0004 and 0.0004, ** P = 0.0034 and as determined by two-tailed, t-test. Error bars represent Mean ± SEM of three replicates. (C) Cytotoxicity was measured by lactate dehydrogenase (LDH) assay from culture supernatants treated with 5 µM of the indicated bispidine conjugates or DMSO. LDH released from cells incubated with detergent buffer was used as 100% LDH release. (D) IC_50_ value for Bisp-W in the indicated cell lines was estimated by measuring viral titers in cell culture supernatants (22 h pi) infected with JEV and treated with the indicated concentration of Bisp-W. Error bars represent Mean ± SEM of three replicates. All the data are representative of experiments performed at least twice with three replicates.

The IC_50_ of BLB for JEV in the above experiments was determined to be around 30 µM (data not shown) and in order to enhance the potency of bispidine conjugates we next synthesized derivatives with varying hydrophobicity in order to determine the effect of hydrophobicity on the inhibitory efficiency. The more hydrophobic tryptophan (Bisp-W), lysine (Bisp-K) and a dipeptide (Bisp-LF) derivative of bispidine were tested against JEV (Figure 3A). All the analogues (Bisp-W, Bisp-LF, Bisp-K) were synthesized by reacting protected amino acid/peptide acid with bispidine using DCC coupling procedure (supplementary information). We assessed the anti-JEV activity of these amino acid conjugates as described above and our initial experiments suggested that these compounds inhibit JEV replication at low micro molar concentrations. We found that the different amino acid derivatives varied in their potency to inhibit JEV production. At 5 µM concentrations, the tryptophan conjugate (Bisp-W) was the most potent inhibitor, which reduced the JEV titers in the supernatant by more than 100-fold (Figure 3B). We verified that none of these compounds affected cell viability by cytotoxicity assay (Figure 3C). Further dose-dependence experiments with Bisp-W and Bisp-LF showed that both the compounds were effective inhibitors in low micro molar concentrations however Bisp-W seemed to be the most potent inhibitor with an IC_50_ value around 1 µM in both N2A and C6/36 cells and as observed in the case of BLB, the IC_50_ value for Bisp-W in Huh7 cells was higher than that observed in neuronal cells (Figure 3D and Figure S1). Since the replication efficiency and kinetics of JEV may vary depending on cell lines, these differences are not unexpected if the drug target/s is/are present at higher amounts under conditions of active viral replication and/or spread in different cell lines.

**Figure 4:**
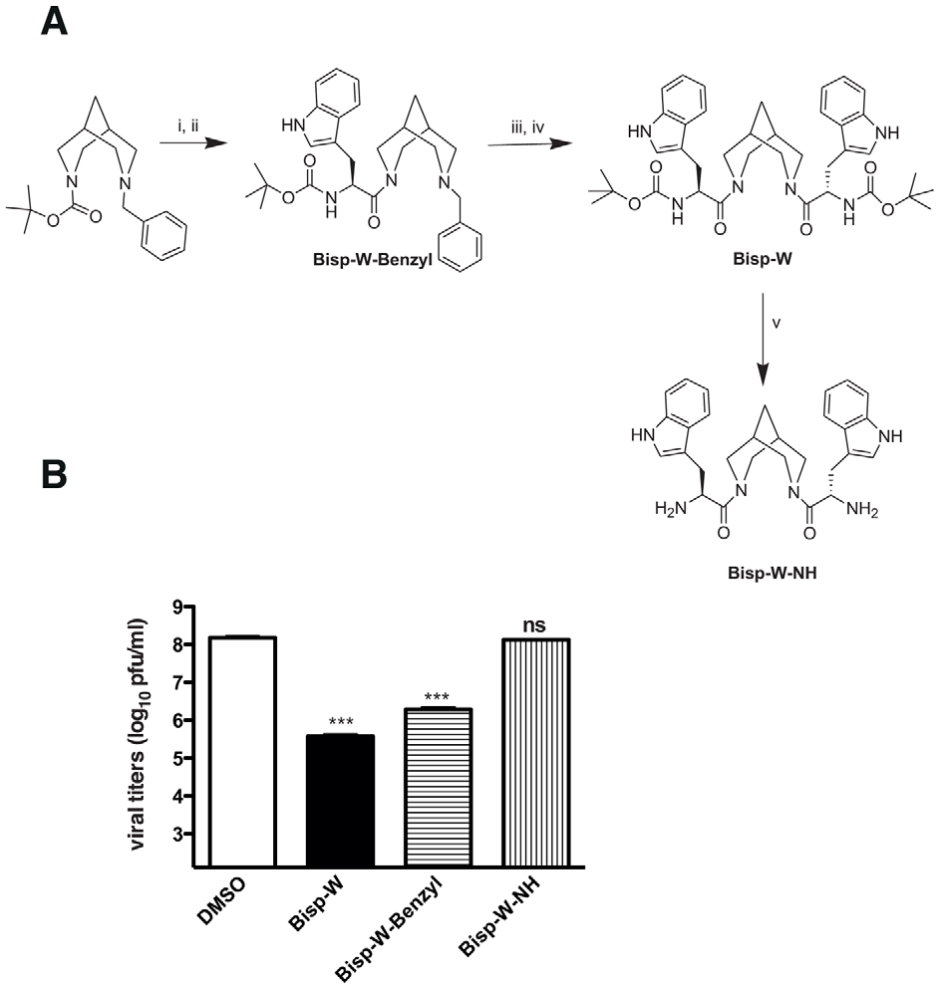
Synthesis of Bisp-W derivatives and its effect on JEV. (A) Structure of Bisp-W derivatives with tryptophan on one arm of bispidine and benzyl on the other (Bisp-W-Benzyl) and with Boc group deprotected (Bisp-W-NH). (B) Viral titers were determined by plaque assay of N2A cell culture supernatants (22 h pi) infected with JEV and treated with 5 µM derivatives of Bisp-W as indicated. *** P = 0.0003 and 0.0004 and ns: not significant (P = 0.425) as determined by two-tailed, t-test. Error bars represent Mean ± SEM of three replicates. Data are representative of experiments performed twice with three replicates.

We further synthesized Bisp-W-benzyl, and Bisp-W-NH and compared the inhibitory efficiency against Bisp-W (Figure 4A). The inhibition studies show that bispidine with one Trp and benzyl (Bisp-W-benzyl) decreased JEV infection by hundred-fold but were less potent compared to Bisp-W. Deprotection of terminal Boc groups (Bisp-W-NH) led to complete loss of antiviral activity suggesting that the hydrophobic terminal (Boc) has a role in the inhibition (Figure 4B).

### Tryptophan conjugate blocks JEV viral RNA replication

**Figure 5:**
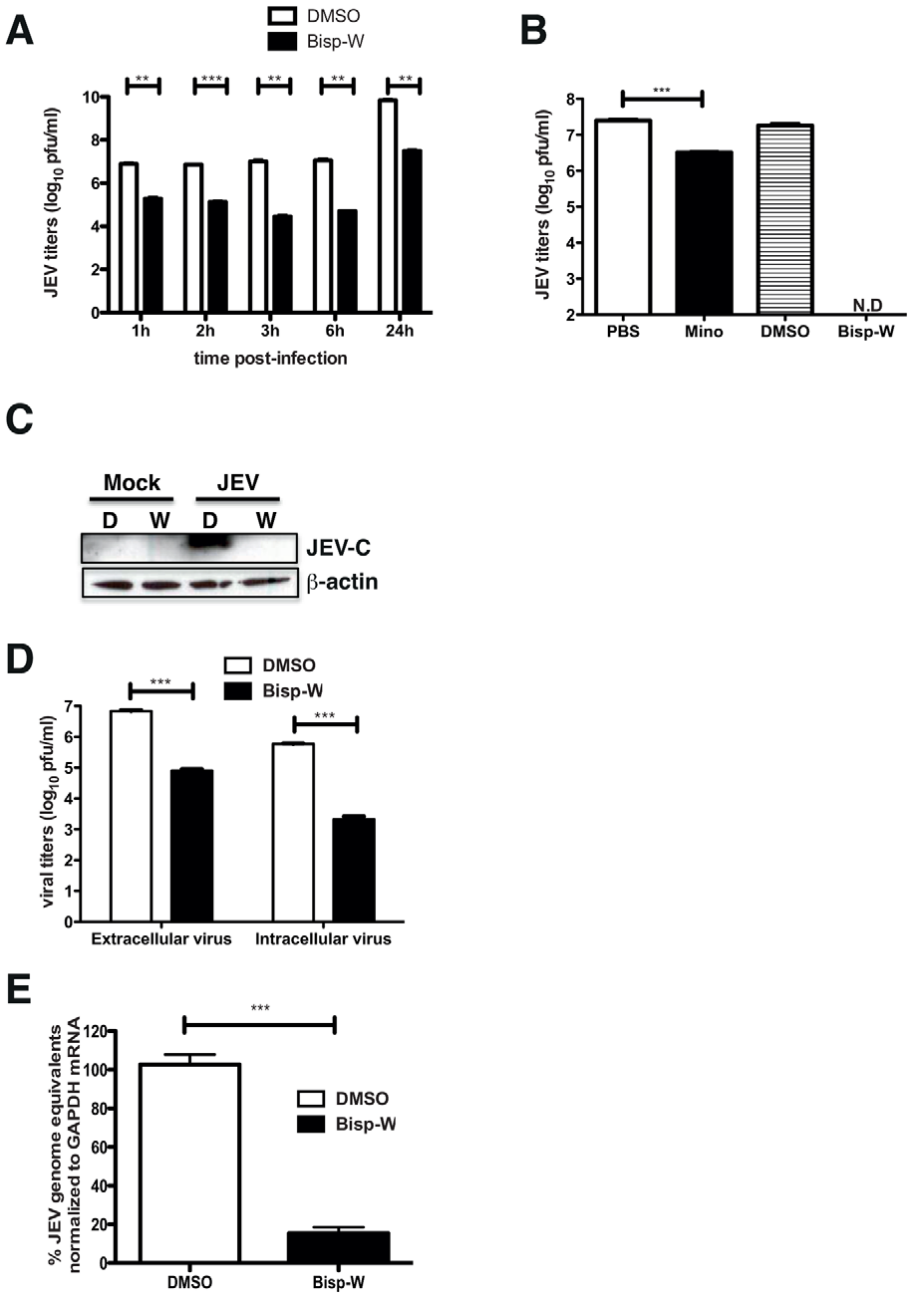
Bisp-W blocks JEV RNA replication. (A) Viral titers were determined by plaque assay of N2A cell culture supernatants (22 h pi) infected with JEV and treated with DMSO or 5 µM of Bisp-W at the indicated time points. Error bars represent Mean ± SEM. ** P<0.005, **** <0001, * <0.01, ** 0.096 and ** <0.001 for respective time points. (B) Viral titers were determined by plaque assay of N2A cell culture supernatants infected with JEV and treated with PBS or 20 µM minocycline and DMSO or 5 µM of Bisp-W as described in materials and methods. Error bars represent Mean ± SEM. *** P = 0.0002. N.D: Not Detected. (C) Western blot analysis of N2A lysates prepared from JEV-infected and DMSO or Bisp-W-treated cells. C- Capsid. β–actin is shown for loading control. (D) Viral titers were determined from supernatants (extracellular) and cell lysates (intracellular) at 22 h pi by plaque assay of samples from N2A cells infected with JEV and treated with 5 µM of Bisp-W at 1 h post-infection. ***P = 0003 and 0.00002 respectively. (E) Total RNA was isolated from N2A cells infected with JEV and treated with 5 µM Bisp-W at 1 h post-infection. JEV genome copy numbers were estimated by quantitative real time PCR normalized to GAPDH mRNA. ** P = 0.0001 by unpaired two-tailed t-test. Error bars represent Mean ± SEM. Data are representative of two or more experiments performed with three replicates.

To further verify that the tryptophan derivatives of bispidine have a similar mechanism of antiviral action, we performed time of addition experiments wherein the compound was added onto the cells post-infection at indicated time-points. We found a 100-fold reduction in the JEV genome levels in Bisp-W treated cells at all time points suggesting that the compound was inhibiting JEV RNA replication and/or later stages of viral life cycle (Figure 5A). We next compared the efficiency of inhibition of Bisp-W with minocycline that has been shown to inhibit JEV previously [8]. We found that, under similar conditions of treatment and infection, minocycline was able to inhibit JEV virus production by about 80%, which is in agreement with a previous report [8] whereas Bisp-W completely inhibited JEV replication and no plaques could be detected in the supernatants (Figure 5B). We next performed western blot analysis of cell lysates prepared from JEV-infected, Bisp-W-treated or mock-treated cells. JEV-capsid protein was readily detected in DMSO-treated, infected cell lysates while Bisp-W-treated samples showed no capsid protein indicating that the block is prior to viral protein translation (Figure 5C). We also measured the extracellular and intracellular virus titers in DMSO or Bisp-W-treated, JEV-infected samples and found a similar 100-fold reduction in both intra- and extracellular virus clearly demonstrating a block prior to virus protein translation and assembly (Figure 5D). We next isolated total RNA from JEV-infected, Bisp-W-treated or DMSO-treated cells and measured the JEV genome copy numbers by quantitative real time PCR. We found that Bisp-W-treated cells had 90% reduction in the JEV genome levels as compared to DMSO-treated cells confirming the block in viral RNA replication (Figure 5E).

### Effect on other encephalitic viruses

**Figure 6:**
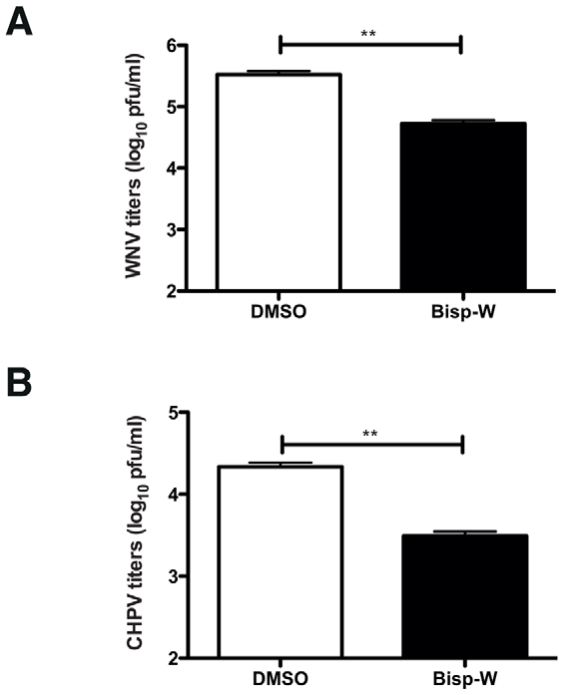
Effect of Bisp-W on other encephalitic viruses. (A) N2A cells were infected with an MOI of 1 pfu/cell with WNV and treated with DMSO or 5 µM of Bisp-W. Viral titers in cell culture supernatants (22 h pi) were determined by plaque assay. (B) N2A cells were infected with an MOI of 0.1 pfu/cell with CHPV and treated with DMSO or 5 µM of Bisp-W. Viral titers in cell culture supernatants (8 h pi) were determined by plaque assay. Error bars represent Mean ± SEM of three replicates. Data are representative of two experiments performed with triplicate samples. **P = 004 and 0.002 respectively as determined by unpaired, two-tailed t-test.

In order to verify the specificity of the antiviral property of Bisp-W, we infected N2A cells with West Nile virus (WNV), which is a closely-related virus within the flavivirus genus and with Chandipura virus (CHPV), a RNA virus from the family *Rhabdoviridae*. Both these viruses are known to be neurotropic viruses causing encephalitis similar to JEV. Cells were treated with DMSO or Bisp-W post virus adsorption and viral titers from the infected culture supernatants were measured at 8 h pi (CHPV) or 22 h pi (WNV). As shown (Figure 6A and 6B), Bisp-W was able to reduce WNV and CHPV titers by 10-fold indicating an altered potency for other encephalitic viruses. This suggests that although Bisp-W is capable of inhibiting other RNA viruses causing encephalitis, the compound is most potent against JEV under our experimental conditions.

### Conformational studies

**Figure 7:**
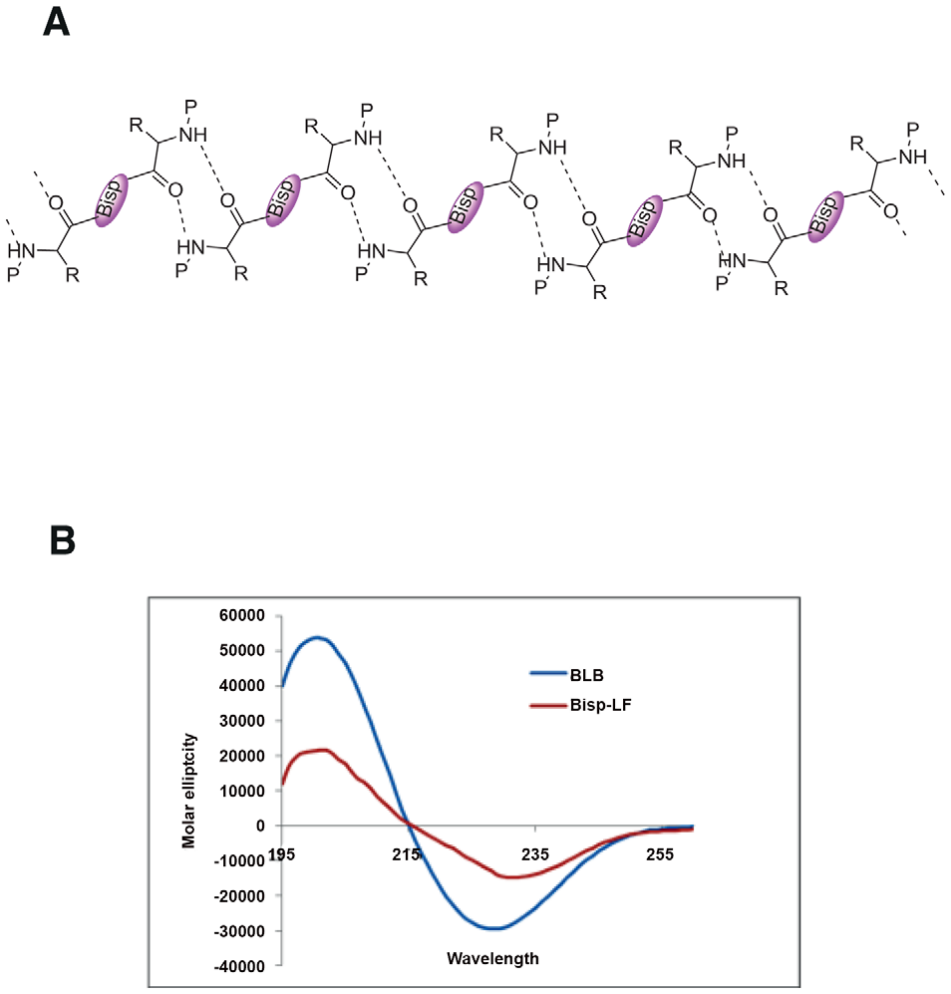
Bispidine conjugates as secondary structure mimetics. (A) Secondary structure showing a β-sheet-like arrangement of BLB in the solid state involving a 10 membered H-bonded ring. (B) CD spectra of 500 µM of Bisp-LF, BLB in MeOH.

The conformation of bispidine derivatives was analyzed by ^1^H NMR, CD, IR and X-ray crystallography. The NMR spectra of all the diimide compounds (Bisp-W, Bisp-LF, BLB and Bisp-K) showed the presence of rotational isomers (syn/anti) as a result of rotation around bispidine CO bond and therefore, is equivalent to proline imide bond (Supporting Information). We calculated the energy barrier of rotation and found to be 80 cal/mol similar to proline cis-trans isomerisation barrier. Both the forms (syn and anti) were present in the chloroform solution indicating a slow exchange. In DMSO, mostly one conformer is detected indicating the influence of solvent polarity in the conformer equilibrium (Supplementary information). The rotation about the bisp CO bond is similar to cis-trans isomerism present in proline-containing peptides. BLB was crystallized from methanol, and the x-ray crystallographic studies unambiguously indicated a β-sheet-like arrangement in the solid state involving a 10-membered H-bonded ring (Figure 7A) [10]. The BLB and Bisp-LF showed a CD with a minimum of 227 nm in methanol supporting β-sheet-like organization (Figure 7B). There are very few examples of templates that can induce β-sheet through intermolecular interactions. All the diimide-linked peptides showed similar CD spectra indicating all of them have β-sheet type conformation (data not shown).

## Discussion

JEV is the leading cause of encephalitic deaths in south and Southeast Asia. A number of earlier reports have identified chemical compounds, either natural or synthetic, that inhibit JEV replication both *in vitro* and *in vivo* [8], [12]–[14]. Minocycline, at 20 µM, was able to reduce the JEV RNA levels in N2A cells by 50% upon a combination of pre-treatment for 1 h prior to infection and for 24 h post-infection and was shown to inhibit neuronal apoptosis induced by JEV. The protective effect of minocycline was also demonstrated in mouse models of JEV infection where 100% of the JEV-infected mice treated with minocycline survived. It was proposed that the protective effect of minocycline was due to its anti-apoptotic and anti-inflammatory activity rather than a direct antiviral action [8]. We show that under our *in vitro* conditions, Bisp-W was more potent than minocycline in inhibiting JEV infection. Non-steroidal anti-inflammatory drugs (NSAID) such as aspirin, indomethacin and sodium salicylate have been shown to inhibit viral production when added post-virus adsorption in mouse N18 cells. However aspirin and sodium salicylate showed inhibitory effects at 5 mM concentrations while indomethacin inhibited JEV titers by 50% at 100 µM [15]. Dehydroepiandrosterone (DHEA), an adrenal derived steroid was also shown to inhibit JEV production at post-entry stages in N18 cells at high micro molar concentrations. DHEA was shown to exert its anti-JEV effect by reversing the inactivation of extracellular signal-regulated protein kinase (ERK), caused by JEV infection, and preventing apoptotic cell death [12]. In another study, peptide-conjugated morpholino oligomer targeting the 5′UTR of JEV genome inhibited JEV replication both *in vitro* and *in vivo* in a suckling mouse model when administered intra-cerebrally [11]. In our study, 5 µM concentration of Bisp-W treatment resulted in 100–1000 fold reduction in JEV titers indicating that hydrophobic amino acid conjugated bispidine derivatives exhibit more potent antiviral activity *in vitro* compared to most of the previously reported inhibitors for JEV.

In the present study we have identified bispidine as a scaffold that in unison with amino acids acts as a potent antiviral compound. The high hydrophobic surface of bispidine can interact with hydrophobic surfaces of proteins and thus may block protein-protein interactions that play a role in JEV replication. Previous reports have suggested that compounds that target protein-protein interactions have a great therapeutic potential due to their high specificity. Understanding the molecular details of protein-protein interactions in viruses have led to the development of a variety of small peptide inhibitors that are capable of blocking such interactions and inhibiting virus infection or replication [4]. We believe that using bispidine as a scaffold to conjugate small peptides could prove to be an additional useful strategy for generating a new class of viral inhibitors. The use of templates to build secondary structure mimetics as potent and effective antimicrobials has been proposed by many early studies [16], [17]. Our x-ray crystallographic studies indicate a β-sheet like arrangement of BLB in the solid state and therefore we speculate that bispidine conjugates can be potentially used for development of specific and potent secondary structure mimetics with anti-microbial activity.

We show that the blockade in JEV replication occurs at the level of viral RNA replication indicating that the possible viral targets could be the viral RNA polymerase NS5 or the RNA helicase NS3. Docking studies are underway with various known structures of flaviviral proteins with bispidine conjugates identified in our study. The identification of the target of bispidine conjugates will help in the rational design of more potent viral inhibitors. Our further investigations will also focus on validating the safety and efficacy of these derivatives in animal models and for human use.

## Supporting Information

Methods S1 **Compound synthesis and purification procedures and NMR spectra indicating purity of the compounds is provided as supplementary methods and figures.**
(DOCX)

Figure S1 **IC50 value for Bisp-LF. IC50 value was estimated by measuring viral titers in cell culture supernatants (22 h p.i.) infected with JEV and treated with the indicated concentration of Bisp-LF. Error bars represent Mean ± SEM of two or three replicates.**
(TIF)
