# Correction: Bispidine-Amino Acid Conjugates Act as a Novel Scaffold for the Design of Antivirals That Block Japanese Encephalitis Virus Replication

**DOI:** 10.1371/journal.pntd.0011917

**Published:** 2024-01-25

**Authors:** V. Haridas, Kullampalayam Shanmugam Rajgokul, Sandhya Sadanandan, Tanvi Agrawal, Vats Sharvani, M. V. S. Gopalakrishna, M. B. Bijesh, Kanhaiya Lal Kumawat, Anirban Basu, Guruprasad R. Medigeshi

After publication of this article [1], concerns were raised about Fig 2. Specifically, in Fig 2D, the DAPI panels for the Mock and DMSO treatment conditions were duplicated.

**Fig 2:**
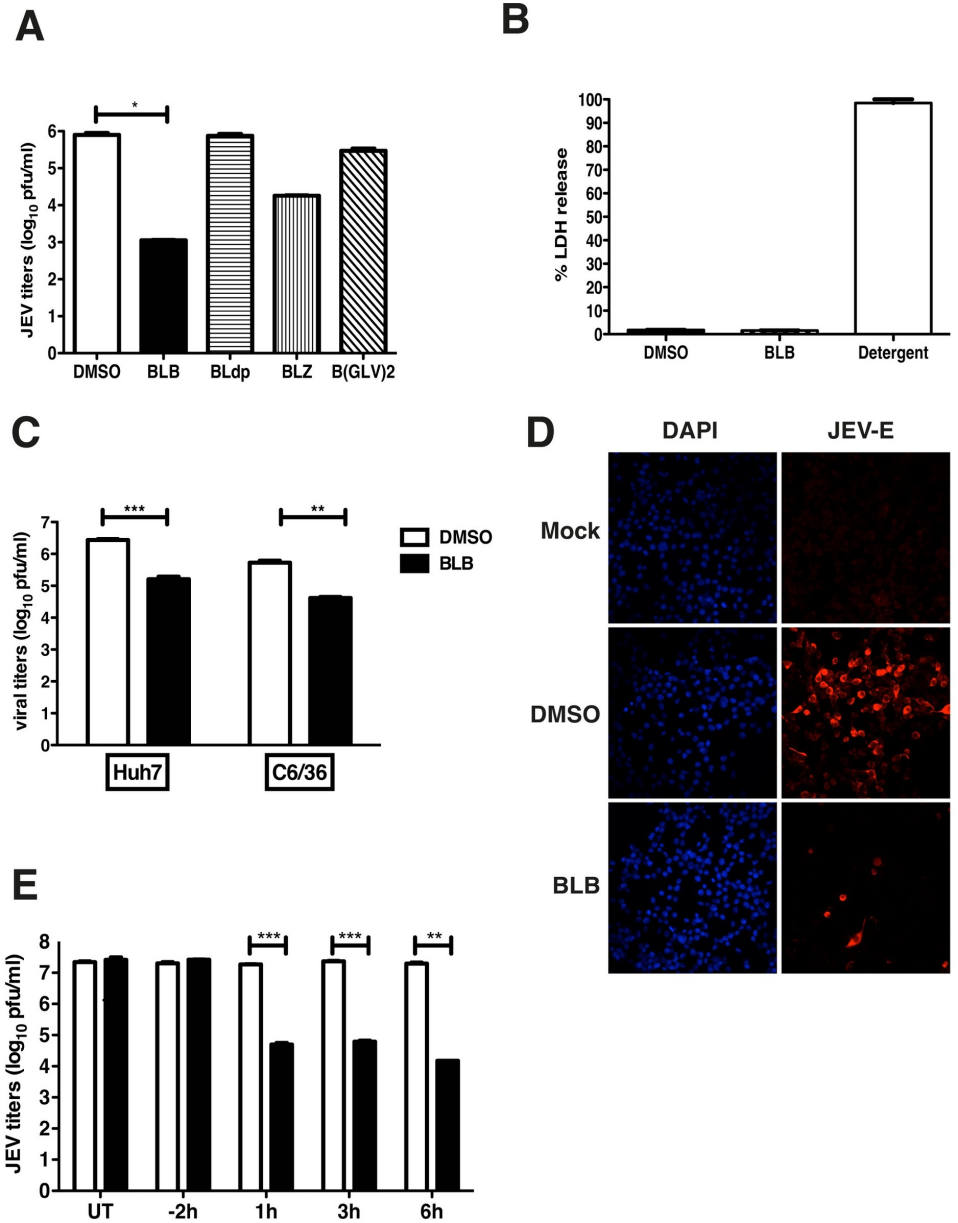
Effect of Bispidine derivatives on JEV infection. (A) Viral titers were determined by plaque assay of N2A cell culture supernatants (22 h pi) infected with JEV and treated with 100 μM of derivatives of bispidine. * P<0.01 determined by two-tailed, t-test. (B) Cytotoxicity was measured by lactate dehydrogenase (LDH) assay from culture supernatants treated with 100 μM of BLB or DMSO. LDH released from cells incubated with detergent buffer was used as 100% LDH release. (C) Viral titers were determined by plaque assay from Huh7 and C6/36 cell culture supernatants (22 h pi) infected with JEV and treated with 100 μM of BLB. *** P = 0.0002 and **P = 0.0041 determined by two-tailed, t-test. (D) N2A cells were infected as above and at 22 h pi cells were fixed and stained with anti-E antibodies followed by alexa 568-conjugated secondary antibodies. Nuclei were stained by DAPI. (E) Viral titers were determined by plaque assay of N2A cell culture supernatants (22 h pi) infected with JEV and treated with 100 μM of BLB at the indicated time points. UT- Untreated. All the data presented are representative of two or more experiments performed with two or more replicates. *** P = 0.0007, 0.0005 and **P = 0.007 as determined by two-tailed, t-test. Error bars in all figures represent Mean ± SEM.

The authors stated that the above concern was due to an error made during preparation of the figures and provided the underlying images for all panels in Fig 2D and a replacement panel to correct the error.

The authors confirmed that underlying data for all panels was still available and that this can be accessed upon reasonable request as per journal requirements at the time of publication.

With this correction, the authors provide a corrected panel for Fig 2. The underlying image data for the corrected Fig 2D (correct DMSO panel from the original experiment) are provided in S1 File.

The authors apologize for the error in the published article.

## Supporting information

S1 File Underlying image data for the corrected Fig 2D (correct DMSO panel from the original experiment). (ZIP)
