# Supplementary material for: Bispidine-Amino Acid Conjugates Act as a Novel Scaffold for the Design of Antivirals That Block Japanese Encephalitis Virus Replication
Source: PLoS Negl Trop Dis. 2013 Jan 17;7(1):e2005. doi: 10.1371/journal.pntd.0002005 (PMC3547849; doi:10.1371/journal.pntd.0002005)
Supplement: Methods S1 — Compound synthesis and purification procedures and NMR spectra indicating purity of the compounds is provided as supplementary methods and figures. (DOCX) [file pntd.0002005.s001.docx]

**Supplementary Methods:**

Compound synthesis and purification

All amino acids used were of L-configuration. unless otherwise stated. All solvents were dried from appropriate drying agent prior to use. Reactions were monitored by thin layer chromatography (TLC). Silica gel G (Merck) was used for TLC and column chromatography was done on silica gel (100-200 mesh) columns, which were generally made from slurry in hexane, hexane/ethyl acetate or chloroform. Melting points were recorded in a Fisher-Johns melting point apparatus and were uncorrected. Optical rotations were measured with a Rudolph Research Analytical Autopol^®^ V Polarimeter; concentrations are given in grams/100 mL. IR spectra were recorded on a Nicolet, Protégé 460 spectrometer as KBr pellets and also in chloroform. ^1^H NMR spectra were recorded on Brucker-DPX-300 (^1^H, 300 MHz; ^13^C, 75 MHz) spectrometer using tetramethylsilane (^1^H) as an internal standard. Coupling constants are in Hz and the ^1^H NMR data are reported as s (singlet), d (doublet), br (broad), br d (broad doublet), t (triplet), q (quartet), m (multiplet). HRMS were recorded with AB Sciex, 1011273/A model using ESI-technique. CD measurements were made using Aviv spectropolarimeter. Quartz cell of 0.1 cm was used for the measurements. All the measurements were done in methanol at room temperature and the peptide concentrations of 100 µM and 500 µM were used. The CD data are reported as molar ellipticity (ME).

Preparation of diimide derivative

To an ice-cooled solution of Boc-protected amino acid or peptide (1.5 mmol) in 65 mL of dry dichloromethane, was added N-hydroxysuccinimide (1.5 mmol), DCC (1.5 mmol) and stirred for 10 min. Bispidine (0.75 mmol) and triethylamine (0.192 mL, 1.5 mmol) were added. The reaction mixture was stirred overnight, filtered and washed the filtrate with 0.2 N H_2_SO_4_, water and saturated aqueous NaHCO_3_ solution. The organic layer was dried over anhydrous Na_2_SO_4_, filtered and evaporated to yield the crude compound. It wasthenpurifiedbysilica gel chromatography. The puritywascheckedby reverse phase HPLC usingacetonitrile/water/TFA as solvent. Gradientmodewasused.

Deprotection of Boc group

To an ice- cooled solution of 1mmol of compound (BLB or Bisp-W) in dry dichloromethane (1 mL) was added TFA (1 mL) and stirred at room temperature for 2 hrs. The reaction mixture was subjected to vacuum to remove CH_2_Cl_2_ and TFA. Redissolved in dichloromethane, washed with sat. sodium bicarbonate solution to afford, BL dp, Bisp W-NH respectively.

**NMR spectra of compounds used in this study**
