# Supplementary figures and images for: Bispidine-Amino Acid Conjugates Act as a Novel Scaffold for the Design of Antivirals That Block Japanese Encephalitis Virus Replication
Source: PLoS Negl Trop Dis. 2013 Jan 17;7(1):e2005. doi: 10.1371/journal.pntd.0002005 (PMC3547849; doi:10.1371/journal.pntd.0002005)

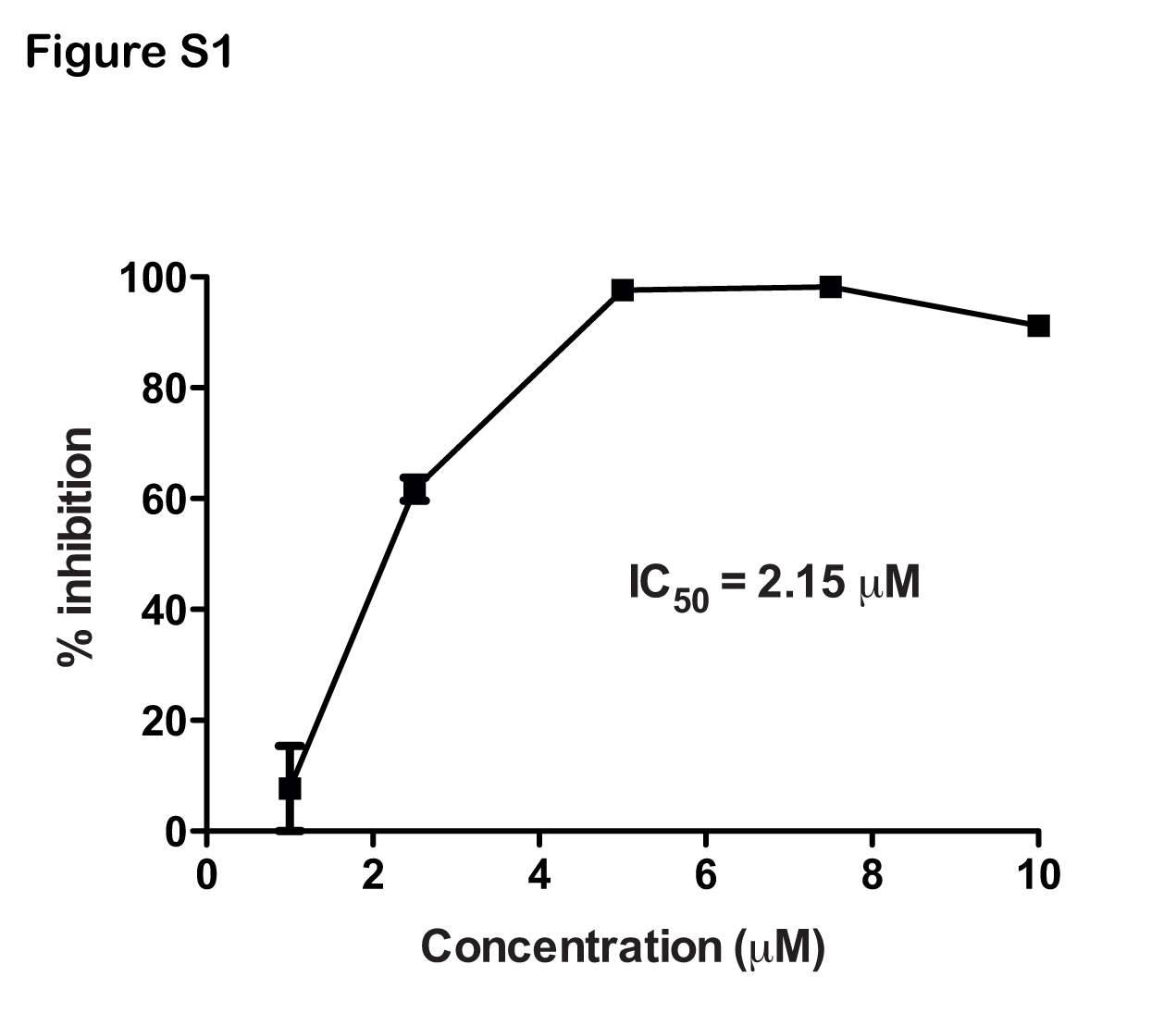

Supplement: Figure S1 — IC50 value for Bisp-LF. IC50 value was estimated by measuring viral titers in cell culture supernatants (22 h p.i.) infected with JEV and treated with the indicated concentration of Bisp-LF. Error bars represent Mean ± SEM of two or three replicates. (TIF) [file pntd.0002005.s002.tif]
